# Patients’ and stakeholders’ experiences of a personalized self-management SUPport program (P-SUP) for patients with type 2 diabetes mellitus and/or coronary heart disease: a qualitative process evaluation

**DOI:** 10.1186/s12889-024-20034-6

**Published:** 2024-09-19

**Authors:** Maximilian Scholl, Jessica Amerkamp, Chloé Chermette, Friederike Frank, Christian Funke, Lisa Giesen, Viviana Haas, Martina Heßbrügge, Lucas Küppers, Larisa Pilic, Frank Vitinius, Bianca Biallas

**Affiliations:** 1 Institute of Movement Therapy and Movement-oriented Prevention and Rehabilitation, German Sports University Cologne, Cologne, Germany; 2 Institute of Psychology, German Sports University Cologne, Cologne, Germany; 3 Institute for Digitalization and General Medicine, Medical Faculty, RWTH Aachen University, Aachen, Germany; 4 Center for Rare Diseases Aachen (ZSEA), Medical Faculty, RWTH , Aachen University, Aachen, Germany; 5 IInstitute of General Practice, Heinrich Heine University Düsseldorf, Düsseldorf, Germany; 6 Institute of Health Economics and Clinical Epidemiology, University Hospital Cologne, Cologne, Germany; 7 Institute of General Practice, University Duisburg-Essen, Essen, Germany; 8 Institute of Family Medicine and General Practice, University of Bonn, Bonn, Germany; 9 Institute of General Practice, University of Cologne, Faculty of Medicine, Cologne, Germany; 10 Department of Psychosomatics and Psychotherapy, Faculty of Medicine, University Hospital and University of Cologne, Cologne, Germany; 11 Department of Psychosomatic Medicine, Robert-Bosch Hospital, Stuttgart, Germany

**Keywords:** Type 2 diabetes mellitus, coronary heart disease, Disease management program, Lifestyle modification, Peer support, Complex intervention, Process evaluation

## Abstract

**Background:**

Chronic diseases such as type 2 diabetes mellitus and coronary heart disease cause medical, social, and economic burdens worldwide. Disease management programs in Germany mostly lack components to improve patients’ self-management and health-promoting lifestyles despite clear guideline recommendations. Therefore, a Personalized Self-Management Support Program (P-SUP) was developed, which includes: (1) peer support groups; (2) telephone coaching; (3) feedback reports and, (4) a web portal. This study aims to explore patients’ and implementing stakeholders’ experiences in the implementation of P-SUP to identify barriers and facilitators to the implementation of the intervention.

**Methods:**

A qualitative study was conducted using face-to-face focus group interviews with participating patients and telephone-based one-to-one expert interviews with implementing stakeholders, involved in the delivery of the intervention. The transcribed interview reports were analyzed using qualitative content analysis, and the contents were categorized according to Donabedian’s Structure-Process-Outcome framework.

**Results:**

A total of six themes among patients (*N* = 35) and five themes among implementing stakeholders (*N* = 32) represent the experiences. The patient themes were: (1) technical conditions, (2) indoor facilities, (3) group composition, (4) acceptance of digital components, (5) supervision and feedback and (6) impact on lifestyle behavior. The themes among the implementing stakeholders were: (1) multiprofessional approach, (2) human resources, (3) patient acceptance, (4) supervision and feedback and (5) impact on lifestyle behavior.

**Conclusions:**

Multiprofessional interventions such as P-SUP appear to be valuable for patients and implementing stakeholders. Although infrastructural barriers made the implementation of peer support and digital patient education difficult, patients rated the exchange with peers and experts on health-related topics positively. The lack of supervision and feedback during the course of the intervention might be compensated by regular telephone coaching. The findings from this study can be used in future studies to guide researchers and implementing stakeholders and to improve the feasibility and effectiveness of complex interventions in different contexts.

**Trial registration:**

The P-SUP study was registered in the German Clinical Trials Register (DRKS) on 16/07/2020 under the registration number DRKS00020592.

**Supplementary Information:**

The online version contains supplementary material available at 10.1186/s12889-024-20034-6.

## Introduction

Chronic diseases, such as type 2 diabetes mellitus (T2DM) and coronary heart disease (CHD), are highly prevalent and have a global impact on public health. The increasing proportion of people with chronic diseases is of particular concern because these conditions limit activities of daily living, require ongoing medical care and are therefore associated with significantly higher health care costs [1, 2]. Due to the enormous burdens on patients and health care systems, there is a need for approaches to effectively manage chronic diseases such as T2DM and CHD.

Two well-known approaches are disease management and the chronic care model [3, 4]. They form the basis of disease management programs (DMPs), which were introduced in Germany in 2002 as a statutory treatment program with a particular focus on primary care. DMPs are standardized chronic care programs based on current medical knowledge and evidence-based guidelines for diagnosis and treatment [5]. They aim to improve the cooperation between different levels of care by specifying tasks and therapies and establishing regular check-ups [6, 7]. Although their effectiveness remains controversial [8], there is evidence that German DMPs have the potential to improve clinical outcomes, as well as quality of life, and decrease mortality [9, 10]. DMP guidelines encourage general practitioners (GPs) to provide advice on self-management and lifestyle modification [11]. However, German DMPs do not include practical components that actively support patient self-management and health-promoting lifestyle behaviors. Until now, infrequent patient education classes [12] every three years and regular consultations by GPs have been the only concrete support. In particular, practical support for a good self-management is an essential strategy to reduce the burden of chronic diseases such as T2DM and CHD [13, 14]. This support needs to include a healthy diet and regular physical activity because of their beneficial effects on T2DM and CHD [15, 16].

Peer support (i.e. support from people with the same or a similar disease) is seen as an effective and resource-efficient way to positively influence the health and self-management of patients with chronic conditions. Systematic reviews have shown that peer support can have a positive effect on clinical parameters and patients’ knowledge about their disease [17]. Moreover, peer support programs have been shown to promote physical activity, increase adherence to exercise programs [18] and improve the dietary habits of patients with chronic diseases [19]. However, the effectiveness of peer support by itself is not undisputed [20], and attending face-to-face programs can be difficult for patients due to comorbidities, scheduling issues, lack of transportation, family and work obligations, or negative feelings about group participation [21–23]. In this context, digital health interventions, such as mobile health applications or devices and websites have emerged as a promising approach for the self-management of chronic diseases. Digital program components have the potential to address barriers to self-management interventions such as disability, lack of mobility, cost, or family responsibilities [24] and have shown improvements in medical outcomes [25–27] and risk-related behaviors such as physical activity and dietary behaviors [14, 28]. In general, personalized feedback on medical parameters or behavioral changes is considered an important component of sustainable lifestyle change [29]. Therefore, additional interventions such as telephone-based health coaching may be an appropriate method for lifestyle behavior change. Especially in vulnerable target groups, it is considered an effective feedback method to continuously adapt an interventions’ content on topics such as exercise and nutrition to the individual needs and goals of chronically ill patients [30].To expand the existing DMP care and to focus on the abovementioned factors, the multimodal complex intervention P-SUP (Personalized Self-Management Support Program) [31] has been developed for patients with T2DM and/or CHD. To gain more insight into the implementation of complex interventions, process evaluations are of increasing interest [32–34]. These are typically conducted using qualitative research during or after the implementation of an intervention to assess and explain outcomes by exploring aspects of implementation, mechanisms of impact and context [32, 34]. To explore how a multimodal intervention, such as P-SUP, can be implemented in German DMP care, this study aims to identify the influencing factors and facilitators for successful implementation and to learn how to overcome barriers. Through insight into patients’ and implementers’ experiences with this process, these findings provide guidance for researchers and practice groups.

## Methods

### Intervention

P-SUP is a randomized controlled trial (10/2019–04/2024) and has been developed and tested in the state of North-Rhine-Westphalia, Germany. A total of 1.006 DMP patients were enrolled and assigned to either the intervention group (IG, *N* = 465) or the control group (CG, *N* = 541). The intervention duration of the IG was 18 months, where the IG received the P-SUP program and the CG received the DMP standard care. Following the 18-month intervention period of the IG, the intervention was also offered to the CG for a period of 12 months. The shortened CG intervention period was due to delays in the intervention start as a result of the Covid-19 pandemic. In the further course of this process evaluation, only IG data will be considered.

The following is a description of the four P-SUP intervention components:

### 1. Peer support groups (PSGs)

Weekly group-based exercise sessions (ES) and regularly scheduled digital expert education classes (DEE). Each PSG consisted of patients with T2DM and/or CHD and a leading patient (PSG leader). The ES were supervised by sports therapists a total of 14 times during the 18-month intervention period. Particularly in the first few weeks, the sports therapist provided regular supervision to provide the PSG and the PSG leader with the exercise content to be performed self-managed in the subsequent ES. GPs were responsible for selecting PSG leaders. During enrollment in P-SUP, GPs asked eligible patients about their interest in participating as PSG leaders. During the selection process, GPs focused oh the patients’ communication skills, organizational skills, and successful disease management. If the patients agreed, they were assigned the role as PSG leader.

### 2. Telephone Coaching (TC)

A specific offer to patients with low health literacy and patient activation (for detailed information, see the TC concept paper [35]).

### 3. Feedback reports (FR)

Quarterly reports for patients containing routine data on medical parameters as a basis for discussing their state of health with their GPs at DMP check-ups. The medical data in the FR were prepared by the Central Research Institute of Ambulatory Health Care in Germany (Zi), included in the reports, and sent to the GPs. The GPs then finally handed the reports to their patients.

### 4: Browser-based web portal (WP)

Provides practical and theoretical contents on exercise, nutrition, and motivation. For detailed information on the P-SUP components, see Additional file I and the study protocol [31].

P-SUP is a multimodal complex intervention that involves patients who participate in the intervention and implementing stakeholders (IST) who deliver the different interventional components (see Table 1). On the patient side, there are participants who are offered all four components of the intervention (TC is only offered to patients with low health literacy/patient activation), participants who are offered three components of the intervention (no TC), and patients who fulfill the role of the PSG leader. On the IST side, GPs were responsible for enrolling their patients into the program and for sending the quarterly FR. Sports therapists were engaged in leading the main interventional component, i.e., the group-based ES. These were complemented by DEE, delivered by experts from various professions, such as medicine, psychology, health or sports sciences. Furthermore, trained telephone coaches with a background in psychology, nurtrition and/or sports sciences conducted the TC.

**Table 1:** Overview of patients and implementing stakeholders

Patients	Role description
Patients with TC	- Patients, who are offered all four intervention components
Patients without TC	- Patients, who are not offered TC
Patient PSG leaders	- Patients, who are intervention participants and PSG leaders
Implementing stakeholders	Role description
General practitioners	- Patient enrollment - PSG leader selection - Sending feedback reports to patients - Discussing feedback reports with patients
Sports therapists	- Supervision of 14 exercise sessions - Development of a group-specific exercise plan - Preparing the PSG for self-managed exercise sessions
Telephone coaches - Nutritionists - Sports scientists	- Conducting 13 telephone coaching sessions - Support patients’ adoption of a healthy lifestyle
Experts - General practitioners - Nutritionists - Sports scientists - Health scientists - Psychologists	- Conducting digital expert education classes - Educating patients in the areas of medicine, nutrition, motivation, and communication/group dynamics

### Study design

This study was conducted in North Rhine-Westphalia, Germany, as part of the pragmatic randomized controlled trial P-SUP. It reports on a qualitative process evaluation of an implementation, conducted from a change-oriented research perspective and framed within the transformative paradigm [36]. While quantitative research methods are used when factual data are needed to answer the research question, qualitative methods are more useful in answering questions about participants’ experiences, meanings, and perspectives [37]. Therefore, a descriptive, qualitative approach was used with two different qualitative methods. Face-to-face focus group interviews with patients were chosen to gain information on patients’ experiences with the P-SUP intervention by predefined topics and free discussions. The social interaction between participants in focus group interviews can lead to lively discussions, making the data collection potentially more comprehensive and meaningful [38]. Due to the limited time resources and geographical distribution of the IST, telephone-based one-on-one expert interviews were conducted to collect information on the implementation of the P-SUP intervention. By reducing the burden on IST [39], the authors hoped to increase their willingness to participate.

The Structure-Process-Outcome (SPO) framework by Donabedian [40] guided the formative process evaluation to identify real-time implementation barriers and facilitators. Because of its flexibility, the SPO framework has been useful in quality improvement initiatives in all clinical settings [41].

### Participants

The target group of this study was patients, who participated in the IG of the P-SUP intervention, as well as IST, who were involved in the delivery of the P-SUP intervention (see Table 1). The study focused on the enrollment of patients who either actively participated in the P-SUP intervention at the time of the study or previously participated in the intervention and withdrew from active participation during the course of the intervention. It also enrolled patients who received additional TC as well as patients who participated as PSG leaders. During the study recruitment process, patients were invited to participate in the study by letter and telephone and IST were invited by email or letter. For data privacy reasons, the invitations were made by the P-SUP Trust Center of the Institute of Health Economics and Clinical Epidemiology (IGKE) at the University Hospital Cologne, which had the sole access to the patient data. To achieve saturation in the number of interviews required, the aim was to conduct 5–8 focus group interviews with patients and 10–15 expert interviews with GPs and sports therapists each [42]. A total of 77/465 (17%) patients were invited to participate. 19/70 patients with TC (27%); 30/395 without TC (8%) and 16/16 trained PSG leaders (100%). In addition, 12 dropouts were invited who had already dropped out of the program at the time of the interviews (12 months after the intervention start). To be eligible for the focus groups, dropouts had to have attended at least one PSG meeting to be able to share their experiences. The authors do not have information on the number of dropouts at the time of the interviews. However, after the 18-month intervention, the dropout rate in the IG was 44% (*N* = 206). It should be noted that the intervention was conducted during the Covid-19 pandemic, which affected the organization and execution of the group meetings and probably led to higher dropout rates.

All patients were randomly selected by the Trust Center. In order to gain the most insightful data possible, the Trust Center aimed to ensure that the invited sample was as gender-balanced as possible, that patients from both DMPs (CHD and T2DM) and different cities in North-Rhine-Westphalia participated, and that patients with different roles within the P-SUP intervention (see Table 1) reported on their experiences. Patient enrollment was stopped when theoretical saturation could be assumed. On the IST side, all GPs (*N* = 137) and all sports therapists (*N* = 29), who participated in the P-SUP trial, were invited. Due to the limited number of experts (*N* = 8) and telephone coaches (*N* = 4), the inclusion of all experts and telephone coaches was intended.

### Data collection

First, focus group interviews were conducted between 09/08/2022 and 30/08/2022 in facilities of different university hospitals (Aachen, Cologne, Düsseldorf, Essen) in North-Rhine Westphalia, Germany. Each interview was conducted by one interviewer and one minute taker. The interviewers were a multidisciplinary team of three with professional backgrounds in sports science, health science, and psychology, who rotated conducting these interviews. Second, expert interviews were conducted between 07/02/2023 and 25/04/2023 by two interviewers with a professional background in sports science. All interviewers were involved in developing P-SUP but did not interact with patients or IST during the intervention.

To participate in the study, an informed consent was obtained from all subjects. Patient demographics were collected by questionnaire prior to the start of the P-SUP intervention and saved at three organizational units (1. Trust Center, 2. Data Warehouse, 3. Multiple Pseudonym Assignment Unit) of the IGKE at the University Hospital Cologne (for more detailed information on data confidentiality, see the study protocol [31]). For privacy reasons, the interviewers did not know which patients were invited to the interviews by the Trust Center and had no access to their data. The demographic data of the patients were made available to the authors for this publication in aggregated form to describe the sample (Table 2). IST demographics were collected by questionnaire after their consent to be interviewed. All interviews were audio recorded and transcribed by a professional external transcription service. References to individuals’ identities in the transcripts were replaced with pseudonyms. Each focus group interview was scheduled to last 90 min, and each expert interview was scheduled to last 30 min.

All interviews were conducted using semistructured interview guides to ensure a degree of flexibility and to ensure the discussion of all relevant areas [43]. The interview guides were based on the SPO framework and tailored to the context and target group (patient or IST; see Additional file II for the interview guides). In this study, structure referred to experiences with material, personal, and infrastructural resources; process referred to experiences with the intervention components; and outcome referred to the perceived impact of P-SUP on health-related behavior.

### Data analysis

A qualitative content analysis [44] was conducted to analyze the interview transcripts. The main categories were developed deductively based on the three dimensions of the SPO framework and the interview guides. The initial review involved careful reading of the transcribed interviews by two researchers, followed by deductive categorization. Notable observations, anomalies, as well as initial ideas for further analysis, were documented as memos for later reference. The results of the first coding phase were reviewed within a larger group of five researchers, and discrepancies were resolved through team discussion until consensus was reached. Subsequently, in a second coding phase, the two researchers subjected one interview transcript to a more in-depth coding procedure to further refine the category system into themes and subthemes (see Table 3). This phase specifically focused on the inductive categorization, where themes emerged from the data itself. Following, the same two researchers then conducted an additional independent validation coding, encompassing approximately 30% of the material, which was subsequently compared and deliberated upon [45]. This method facilitated the examination of consistency and the identification of discrepancies, which were subsequently discussed between the researchers, resulting in adjustments to the category system. Both researchers then proceeded to code the entire transcript material [46]. Upon revisiting the category system, further refinements were discussed and ultimately implemented in consultation with the other participating researchers [45]. The data analysis was conducted using MAXQDA (version 22). To this end, the transcribed interviews were uploaded to MAXQDA and, from that moment on, exclusively processed and analyzed within the software. In addition to controlling and adjusting the imported transcripts, the program was used to develop and establish the category system, perform the codings by the two researchers, and verify their consistency. In the context of the analysis, particular emphasis was placed on utilizing functions such as lexical search, the visual tools (Code Matrix Browser), and cross-tabulations [46]. This study was structured using the standards for reporting qualitative research (SRQR) checklist [47] as guidance.

## Results

41/77 invited patients agreed to participate (53%), of whom 35 (45%) attended the focus group interviews. 14 patients (18%) who were approached were not interested in participating, and the remaining 22 patients (29%) indicated that they were unable to attend any of the focus group interviews due to scheduling conflicts or a lack of mobility. At the end, 14/395 patients without TC (4%), 11/70 patients with TC (16%), and 5/16 PSG leaders (31%) attended the focus group interviews. Of the invited dropouts, five patients with at least one PSG meeting participated. For the IST, 18/137 GPs (13%) gave their consent and participated, 9 GPs (7%) declined participation and 110 GPs (80%) did not respond. 12/29 sports therapists participated (41%) and 17 did not respond (59%). 7/8 experts (88%) and 3/4 telephone coaches (75%) gave their consent and one expert (12%) and one telephone coach (25%) did not respond. At the end, a total of 67 participants (35 patients; 32 IST) participated in this study (see Table 2). The average duration of the seven focus group interviews was 98.45 min (SD = 11.73) and 32.38 min (SD = 13.09) for the 32 expert interviews.

**Table 2:** Participant characteristics (*N* = 67)

	Patients (*N* = 35)	Implementing stakeholders (*N* = 32)
Age (years), mean (SD)	62.7 (7.9)	49.6 (14.7)
**Gender**,** n (%)**		
-Female	14 (40)	15 (47)
-Male	21 (60)	17 (53)
BMI (kg/m²), mean (SD)	33.4 (6.5)	N/A
**DMP affiliation**,** n (%)**		
-DMP CHD	5 (14)	N/A
-DMP T2DM	23 (66)	N/A
-DMP CHD/T2DM	7 (20)	N/A
**Migration background**,** n (%)**		
-Yes	6 (17)	N/A
-No	28 (80)	N/A
-Unknown	1 (3)	N/A
**University entrance qualification**,** n (%)**		
-Yes	14 (40)	N/A
-No	19 (54)	N/A
-Unknown	2 (6)	N/A
**Employment status**,** n (%)**		N/A
-Employed	14 (41)	N/A
-Retired	16 (47)	N/A
-Housework	1 (3)	N/A
-Looking for a job	1 (3)	N/A
-Unemployed	2 (6)	N/A
-Unknown	1 (3)	N/A
**Patient role**,** n (%)**		
-Patients with TC	11 (31)	N/A
-Patients without TC	14 (40)	N/A
-Patient PSG leaders	5 (14)	N/A
-Patient Dropouts	5 (14)	N/A
**IST role**,** n (%)**		
-General practitioner	N/A	10 (31)
-Sports therapist	N/A	12 (38)
-Expert	N/A	7 (22)
-Telephone coach	N/A	3 (9)

SD = Standard deviation; N = Population size; BMI = Body mass index; N/A = not applicable; DMP = Disease Management Program; CHD = Coronary heart disease; T2DM = Type 2 Diabetes mellitus; IST = Implementing stakeholders

Six subthemes represent patients’ experiences of the study intervention, and five themes were identified among IST, which were assigned to the SPO framework dimensions structure, process or outcome (see Table 3).

**Table 3:** Main themes and subthemes

Framework dimension	Main themes (deductive)	Subthemes (inductive) Patients		Subthemes (inductive) IST	
Structure	- Material resources - Personal resources - Infrastructural resources	1)	Technical conditions	1)	Mulitprofessional approach
		2)	Indoor facilities	2)	Human resources
Process	- PSG meetings * - Telephone coaching - Feedback reports - Web portal - Improvement suggestions	3)	Group composition	3)	Patient acceptance
		4)	Acceptance of digital components	4)	Supervision and feedback
		5)	Supervision and feedback	/	
Outcome	- Perceived impact of P-SUP	6)	Impact on lifestyle behavior	5)	Impact on lifestyle behavior

* Questions about PSG meetings included exercise sessions and digital expert education classes

For a better overview, the results of the patients are presented first, followed by the results of the IST.

### Patients

#### Technical conditions

P-SUP provided patients with two digital components: the DEE and the WP. Some patients expressed that the technical requirements for using these digital intervention components were not met by themselves or within the group. Especially for the implementation of the DEE, a lack of technical affinity or equipment proved to be a barrier. Reasons for this were a lack of interest in engaging with new technologies and the age of the target group.

*„[In the DEE] we have the following problem: you have to have a laptop*,* and in our case I think we only have three [participants with a laptop]. […] You cannot ask an 89-year-old to get a laptop.”* [Focus group interview 3; Patient 3].

As a result, some PSGs met face-to-face for DEE and used a group member’s or sports therapist’s device. Because of these challenges, several patients would have preferred face-to-face meetings with the experts or access to facilities equipped with appropriate technology (e.g., large screen, laptop).

### Indoor facilities

Due to contact restrictions during COVID-19, indoor ES were not allowed at the beginning of the intervention. When the restrictions were loosened and indoor meetings were allowed, the PSGs could decide whether to find an indoor location or continue to meet outside. Patients who had access to a suitable location particularly appreciated the ability to respond flexibly to weather conditions and seasonal changes. In addition, some PSGs used the facilities provided to hold DEE when the technical resources within the PSG were insufficient. However, for organizational reasons, not all PSGs had suitable facilities. Especially participants in the drop-out group reported a lack of indoor facilities to hold group meetings (e.g., ES). For participants in this target group, a lack of facilities was one of the reasons for dropping out. The resulting barriers included a lack of sanitary facilities, inappropriate outdoor walkways, and dependence on weather and light conditions.

*„From the very beginning we had meetings outside in all weather. It was cold*,* it was raining*,* so it was not nice. There was no room either*,* so I said I do not have to walk around in the dark*,* in total darkness […]. I do not need to do that to myself.“* [Focus group interview 2; Patient 5].

### Group composition

As far as the group meetings are concerned, patients particularly appreciated the opportunity to engage in discussions with peers with similar medical backgrounds. The exchange of ideas and experiences on topics such as diet and medication were positively evaluated by several respondents. The resulting peer support was perceived as a motivating factor.

*„Most importantly*,* you are among like-minded people. You can exchange ideas. One has a tip*,* the other has a tip. […] and I think that is much better than being on your own.“* [Focus group interview 4; Patient 4].

However, small group sizes and heterogeneity within some PSGs were identified as barriers to the implementation. In particular, significant differences in underlying medical conditions and physical fitness levels were repeatedly viewed negatively.

*„As far as the composition of the group is concerned*,* I actually imagined that I would meet people with the same disease as me. It makes sense to me that diabetes and heart diseases are treated in the same way*,* that the same thing is good for everyone. But in fact*,* I do not benefit from other patients now.“* [Focus group interview 6; Patient 1].

### Acceptance of digital components

DEE on nutrition were rated very differently because patients had different levels of prior knowledge. Some respondents criticized that the topics were already well known. Overall, some patients found the content too theoretical, and there was a desire for more practical advice, such as cooking classes. DEE on communication/group dynamics were rated as the least relevant topic for patients and several respondents saw no need for training on these topics. The DEE on motivation and medicine, on the other hand, were rated positively by all patients. In particular, the ability to discuss medical issues with a medical expert and the opportunity for open discourse were highlighted as positive aspects.

*„However*,* overall*,* that meeting with the [medical] expert*,* I found it truly life changing*,* it was just on a level that I found meaningful. He was very well prepared and took individual questions very seriously. So that was something where I can say that I personally totally benefited from it*,* I loved it.“* [Focus group interview 6; Patient 1].

In addition to the DEE, the WP could also be used as a source of patient education. Several respondents reported that they rarely or never used the WP. Patients cited lack of interest and time as the main reasons for infrequent use. Other respondents also cited lack of information about the existence of the WP as a barrier to use. However, patients who actively used the WP rated the exercise videos and recipes as positive.

### Supervision and feedback

Initially, the ES were supervised weekly by a sports therapist, and over time, the patients had to do the ES self-managed and the frequency of supervision decreased. The majority of patients felt that the interval between the supervised ES with a sports therapist was too long. Specifically, to adequately prepare for self-managed ES, patients expressed a desire for more supervision by a sports therapist. In addition, the supervised ES were perceived as more effective, structured, and motivated.

*„Alone [without a sports therapist]*,* I would say we do it half-heartedly*,* our mind is not on the job. When we’re supervised*,* or under supervision*,* whatever you call it*,* we have a completely different mindset.“* [Focus group interview 5; Patient 4].

Several patients reported that the self-managed ES sometimes did not take place at all or that the PSGs were often overwhelmed without external supervision. A lack of movement competence and the fear of performing exercises incorrectly were repeatedly cited as barriers to self-managed ES. The presence of a trained PSG leader was often not sufficient to conduct the self-managed ES. Some PSG leaders themselves felt that the level of responsibility was too high, and PSG members often felt that their PSG leaders were not qualified to lead the group.

*„Yes*,* but [the PSG leader] was too good for this world. He’s a nice guy*,* but as a group leader you also need someone to make an announcement […]. However*,* then there was a ten-minute discussion*,* […] so if someone had made a clear announcement*,* “This is how it works”*,* then certain things could have been avoided.“* [Focus group interview 2; Patient 1].

The FR were intended to provide feedback on medical parameters to be discussed with the GP. While the underlying idea of FR was positively evaluated by some patients, other respondents indicated that they had not received them at all. As a result, patients expressed a desire for more feedback on their individual performance and medical parameters from their GPs.

Conversely, patients who participated in TC expressed high satisfaction with the continuous feedback provided. Through individualized support and the opportunity for collaborative goal-setting and monitoring, these patients reported increased motivation to implement health-related goals. In particular, practical advice on diet was well integrated into their daily lives.

*„The whole program would not work for me without the telephone coaching*,* because that is individual counseling. It is a compensation for fluctuations in motivation. I have had experiences where my motivation has been a bit low and then I have got truly interesting input […].“* [Focus group interview 5; Patient 1].

### Impact on lifestyle behavior

Several patients (20/35, 57%) reported that the intervention had a positive impact on their daily health-related behaviors or even improved medical outcomes. Respondents reported that they became more conscious of what they ate. Specifically, respondents reported that the intervention had influenced their daily exercise patterns, leading them to walk or bike more often. Other patients reported that they had enrolled in other exercise classes since participating, such as rehabilitation sports or aquatic gymnastics.

*„Since I started doing P-SUP*,* I still go to sports on Mondays and Fridays*,* which means rehabilitation sports and machine training. I did not do that before. I always signed up but did not go. […] and I do everything within 5 kilometers by bike*,* not by car.“* [Focus group interview 4; Patient 1].

The results of the expert interviews are presented below.

### Implementing stakeholders

#### Multiprofessional approach

On a structural level, several GPs and medical experts saw an increased need for multiprofessional approaches to patient care due to the increasing lack of time in medical practices. According to the respondents, time constraints in medical care are the reason why relevant issues such as exercise and nutrition are not sufficiently addressed.

*„You have to consider that in regular care*,* when you go to the GP or the specialist*,* they usually have five to seven*,* if you are lucky*,* ten minutes for you. You can not talk about the everyday topics in that time.“* [Expert interview; Expert 6].

Only one medical expert was critical of the involvement of other professions to intervene in the treatment of patients. The respondent was rather sceptical about multimodal treatment and considered the involvement of other professions as potentially disruptive to the doctor-patient relationship.

### Human resources

Telephone coaches, sports therapists and experts considered the workload to be adequate. However, organizing the TC sessions took more time than expected for the coaches, as some patients had frequent hospital admissions due to comorbidities, which meant that appointments had to be rescheduled.

According to the GPs, enrollment of patients needed the most effort. Thus, the GPs felt that the internal resources of their practices were sufficient, as no additional work was needed during the intervention. GPs who still reported personnel barriers to implementation attributed the difficulties primarily to inadequate communication within their practice team.

### Patient acceptance

Several GPs reported difficulties enrolling patients. Patients who could be persuaded to participate were often those who were already actively managing their health, and patients with low health literacy and low patient activation were particularly difficult to enroll. This was mainly due to a lack of interest on the patient side to be actively involved in the management of their disease.

*„That was to be expected*,* because a certain number of patients simply focus on their own responsibility*,* want to do something and are happy to accept what is offered. A slightly larger proportion - and this also applies to different indication areas - like to passively accept medicine and be treated […] and are not very active or proactive.“* [Expert interview; GP 1].

According to the sports therapists and telephone coaches, the patients were generally open to the intervention components. Only the experts reported very different experiences with the DEE. While mostly positive experiences in the areas of medicine, motivation, and nutrition were reported, problems were more frequent in the topic of communication/group dynamics. According to the experts, this topic was used by patients to vent their frustrations about problems within the PSG or general dissatisfaction.

*„What I can report from the [DEE] on group dynamics or communication […] was that it was a topic where the patients first of all*,* I think*,* misunderstood a little bit what it was about*,* and since we were very often confronted with topics […] such as that the groups are not taking place at all*,* that there are structural difficulties for the patients*,* so there was a lot of frustration that was let out from patient side.“* [Expert interview; Expert 2].

### Supervision and feedback

Similar to the patients, most of the sport therapists expressed a desire for a higher frequency of supervised ES to prepare the PSG for self-managed ES. In general, the motivation for self-managed ES varied widely. While some PSGs met regularly without the sports therapist, others did not meet without supervision. According to the sports therapists, the successful implementation of self-managed ES is highly dependent on a dedicated PSG leader, who organizes and takes responsibility.

*„I would find it difficult […] if you have a group where nobody feels responsible for it. I do not know if there’s always the motivation to actually go to the meetings. Of course*,* if you know that there is [a PSG leader] who has a plan*,* who knows what needs to be done*,* […] it is easier than if none of the participants feel responsible.“* [Expert interview; Sports-therapist 1].

Most GPs reported a lack of feedback between them and their patients, since they rarely or never discussed FR or P-SUP in general with their patients during check-ups. This was partly because the intervention was forgotten over time or because the practice staff were sometimes unable to assign the FR. Other GPs saw no added value in the FR, as the content would be discussed during check-ups anyway.

Like the patients, the telephone coaches saw TC as a promising option for providing ongoing feedback to patients and contributing to long-term changes in health-promoting behaviors. Especially for patients who could not fully participate in the ES due to physical limitations, TC was a useful method to maintain the current status or to discuss further options for incorporating physical activity into patients‘ daily lives.

*„There were also [patients] who*,* during the course of the programme*,* stopped attending the group and only took part in the telephone coaching*,* […] but still looked for other sports groups and where P-SUP was the initial spark to actually do it.“* [Expert interview; Telephone coach 2].

### Impact on lifestyle behavior

None of the GPs reported a positive impact of the intervention on their patients. One reason given was that patients were not knowingly identified as P-SUP participants during check-ups. Another reason given was that the target group reached was already active, which made the achievement of positive changes more difficult.

*„There were no patients left in the project […] where the adjustment of the metabolic parameters was truly difficult. Of course*,* it would have been exciting to have someone who has not been able to influence the HbA1c for years and then suddenly the project makes a difference. […] However*,* they were not there either.“* [Expert interview; GP 2].

On the other hand, several sports therapists (8/12, 67%) reported improvements in patients‘ fitness or mobility. One sports therapist reported that patients joined rehabilitation sports classes and continued to exercise under its guidance. Another sports therapist reported that one PSG booked an indoor facility on a long-term basis and continued to meet for ES after the intervention.

## Discussion

To help design successful interventions for patients with T2DM and CHD, it is important to learn as much as possible about the intervention components that are most effective and engaging for patients and feasible for IST. This qualitative evaluation of a peer support program for people with T2DM and CHD explored the experiences of patients and IST to understand the most and least helpful factors and the strongest facilitators and barriers to successful engagement.

Despite the acknowledged benefits of adopting a healthy lifestyle in the management of chronic diseases and clear recommendations to integrate lifestyle counseling in primary care [48], the integration remains a practice that is not widely observed [49]. A large body of empirical research supports the assertion that time constraints and a lack of expertise among GPs are often cited as significant barriers to the effective integration of lifestyle counseling into primary care [50–52]. In Germany, primary care practitioners have 8.9 minutes of treatment time per patient during a routine visit [53]. This time constraint often results in stress and an inability to provide patients with the attention they need [54]. Multiprofessional interventions, such as P-SUP, might be a solution to this challenge, as they involve a range of professional expertise and can potentially increase the capacity of practices and bring benefits for patients [55]. GPs in this study reported that the implementation of P-SUP needed no additional time, with the exception of the enrollment process. This favorable finding is likely due to the multiprofessional nature of P-SUP and the fact that the intervention did not alter the duration or frequency of routine check-ups. Despite the small amount of time required in practices, both patients and GPs reported that the FR or P-SUP itself were rarely if ever discussed during check-ups. GPs often cited internal communication problems or the fact that the intervention was forgotten after the recruitment phase. This may be due to the lack of time in primary care described above. However, continuous feedback is essential for the success of peer support programs [56, 57] and a crucial factor in promoting healthy lifestyles [29]. The multiprofessional approach of P-SUP could also be a suitable solution here, as patients receive additional feedback from the telephone coaches, sports therapists and experts within the DEE. The relevance of this feedback is also reflected in the patients’ feedback, who had a very positive experience with the DEE on medical topics and the TC. These components may be an adequate compensation for GPs’ limited time resources. In particular, regular and individualized feedback from trained telephone coaches may be valuable for patients in such programs. Combined with PSG activities such as ES, where a group consensus must be reached, TC also provides flexibility for patients who do not benefit from group meetings, e.g., due to health reasons or difficulties reaching the PSG venue. It offers the opportunity to tailor the content to individual needs, goals and circumstances, which can improve the quality of care for patients with chronic conditions [30, 58, 59]. Although TC cannot replace doctor-patient interactions to discuss medical issues, it can support patients in adopting healthy lifestyles and motivate to maintain these behaviors. Therefore, TC should be offered to all patients, as it is likely that not only patients with low health literacy can benefit from this ongoing coaching.

The above identified lack of feedback was also evident in the implementation of the ES. Regarding self-managed ES, patients and sports therapists agreed that the number of supervised ES was too low and that patients needed more support. Accordingly, several sports therapists have expressed that a more flexible approach to supervised and unsupervised ES would have been more beneficial for some PSG. Thus, there is the option to provide less frequent supervision to groups particularly inclined towards physical activity compared to those facing challenges in implementing self-managed ES. However, that the absence of the sports therapist probably led to lower adherence in some groups is consistent with findings from reviews comparing adherence to professionally supervised and unsupervised ES [60, 61]. As in the present study, respondents cited a lack of own expertise as well as a lack of feedback from the sports therapists as reasons for low adherence to self-managed ES. To provide patients with a sense of feedback and support during the unsupervised phases, the sports therapist should assign specific exercise tasks for the self-managed ES and require patients to provide regular feedback on their execution, for instance, through the forum function of a WP. This approach could increase the level of feedback and the sense of supervision without increasing contact hours.

To foster consistent adherence and to facilitate group coordination in the sports therapist’s absence, each PSG was designated to be led by a trained PSG leader. In this study, the presence of a trained PSG leader did not contribute to regular adherence in all groups. While the sport therapists emphasized the importance of having a skilled PSG leader to monitor group communication and provide some sort of guidance during the ES, the patients and PSG leaders themselves were rather sceptical of the PSG leader role. It should be critically noted here that not all PSGs had a trained PSG leader. As mentioned in the “Participants” chapter, there were a total of 16 trained PSG leaders, all of whom were invited to participate in the interviews. The reason for the absence of trained PSG leaders in some PSGs was mainly due to difficulties in recruiting suitable patients for this role. As a result, in some groups, patients volunteered to take on organizational tasks without receiving training. This may also be an important reason for the partially low adherence in the PSGs. However, studies have shown, that the presence of a PSG leader can encourage physical activity [62], increase adherence to ES [18], and improve functional, psychosocial, and metabolic outcomes [63, 64]. In this study, some patients considered the PSG leaders to be unqualified, and PSG leaders sometimes felt overwhelmed by their role. An insufficient number of PSG leader training sessions may be a possible explanation for the mentioned challenges. In this study, PSG leaders underwent four half-day training sessions, a frequency comparable to or even exceeding that reported in studies demonstrating satisfactory adherence [63] and the effectiveness of peer-led interventions [63, 64]. Nonetheless, it is notable that the emphasis of these trainings sessions in the mentioned studies was primarily on the exercise components, whereas the preparation for the exercise components in the P-SUP training took up only about a quarter of the time. Therefore, in the future, these training sessions should focus more on the execution of physical activities, as the ES constitute a major part of the intervention. Moreover, the content of the ES was only partially standardized. While all sports therapists were provided with exercise manuals and resistance bands, only the warm-up and cool-down routines were standardized. The flexibility to tailor the ES according to the potential heterogeneity of the PSG appears suitable; however, this may have resulted in situations where the exercises were too challenging for certain patients to reproduce during self-managed ES. The abovementioned peer-led physical activity programs had standardized exercise plans [63, 64] and partly included instructional videos [64]. Although the P-SUP WP included exercise videos, only the standardized warm-up and cool-down were congruent with the content of the ES. A clear structure for the supervised and unsupervised ES and supporting video material might facilitate the implementation of the self-managed ES. Nevertheless, it remains questionable whether self-managed ES can be performed by a PSG without the ongoing supervision of a sports therapist. It seems advisable to have a sports therapist in a continuous support role during the first weeks of the intervention to fully prepare PSGs and PSG leaders for self-managed ES.

One of the main intervention components is peer support, which is considered a potentially effective [17, 65] and resource-efficient [66] approach to chronic disease management, enabling peer-to-peer communication between patients with the same or similar conditions. In this study, the majority of patients were positive about the concept of PSGs. In particular, the ability to share information about disease-specific issues, including medication and diet, was seen as beneficial. However, the group composition plays a critical role in successful implementation [67, 68]. Patients in this study identified a high degree of heterogeneity within the PSG as a major barrier. A degree of uniformity in terms of underlying diseases and physical ability is conducive to facilitating health-related discourse and group-based activities such as ES. It is reasonable to assume that a shared medical condition facilitates this bond, but is not sufficient in itself to create closeness in a PSG. Presumably, some patients were too different in terms of their disease experiences and challenges, personal and social characteristics, and cultural values and lifestyles. However, these factors are fundamental to creating a sense of connectedness within a PSG and, conversely, can be perceived as barriers [67]. A comprehensive pre-screening process for segmentation based on personal, social, and physical characteristics, along with clearly defined inclusion and exclusion criteria for participation in a PSG, could increase the degree of homogeneity and significantly improve group dynamics. Patients who may not be suitable or willing to participate in a PSG could still benefit from the other P-SUP components such as TC or the WP, without being placed in a group situation in which they might feel uncomfortable. The resulting improved group dynamics might potentially facilitate the implementation of the group-based ES and DEE, and could have a positive impact on the effectiveness and sustainability of a PSG. Patients also cited the small group size of 2–4 participants as a barrier to the creation of a positive group dynamic. Group sizes of 4–6 patients were originally planned. The small group sizes may be due to the relatively high dropout rate of 44% in the IG. The high rate may be partly due to the impacts of the Covid-19 pandemic and the associated contact restrictions in Germany. At times, these restrictions allowed only people vaccinated against Covid-19 to meet in groups of a certain size. Because of their vaccination status, fear of infection, or the unpredictability of events, more patients than expected may have dropped out.

As a source of information in the areas of medicine, exercise, nutrition, motivation and psychology, patients were offered DEE and a WP. Due to the COVID-19 pandemic, all education classes were delivered digitally. The technical resources of the patients emerged as a significant barrier for the use of digital components. In particular, the high average age of the target group was seen as a reason for inadequate digital literacy and a frequent lack of technical equipment. These findings are in line with previous studies that found a negative correlation between patient age and the use of eHealth [69, 70]. For the delivery of education classes, patients in this study would have preferred face-to-face contact over internet support. Face-to-face delivery can avoid technical difficulties and build further trust and accountability within a PSG [71]. Therefore, future studies should at least allow for a hybrid solution. With a more flexible format, technology-related concerns could be addressed, thereby reducing barriers to participation. Despite the barriers described, digital components for patient education should continue to be offered due to their proven positive impact on patient health, their potential to bridge geographic barriers, and their time and cost effectiveness [72–74]. Moreover, future patients are growing up with eHealth. It is therefore unlikely that technical affinity and equipment will be a persistent challenge. In terms of DEE content, patients predominantly rated the topics of medicine and motivation positively, while the topics of nutrition and communication/group dynamics were perceived quite differently. The consistently favorable assessment of the medical DEE may result from patients’ limited time to inquire with their GPs during usual care. The DEE afforded patients the chance to pose medical queries, enabling doctors to provide tailored responses. Conversely, the varied reception of the DEE concerning nutrition might be attributed to its sensitive nature among patients, who may harbor heightended confusion due to diverse recommendations and a lack of dietary consensus [75]. Therefore, a more practical approach to nutrition, such as practical cooking sessions, was requested by some patients and should be considered in the future. The DEE on communication/group dynamics were viewed with skepticism. The experts themselves suspected that this topic served as a catalyst for expressing frustration about possible shortcomings of a private nature or within the PSG. Patients found this topic to be of little interest and questioned its relevance. Some patients noted that they had sufficient expertise in the filed of communication through their own professional experience and therefore did not training. However, since the topics of communication and group dynamics are still relevant to the success of a PSG, these topics may be included exclusively in the PSG leader training in the future.

Patients, in particular, reported a positive impact of the P-SUP intervention on their exercise and dietary behaviors. While some PSGs met multiple times per week during the intervention for group exercise, other patients increased their physical activity by using their bicycles more frequently in their daily lives or enrolling in additional exercise classes during the intervention period. Since the participation in a PSG [18, 62, 63, 76], the TC [30, 77], and the digital P-SUP components [78–80] might have had a potentially positive impact on patients’ lifestyle, it remains unclear which component was the most effective. However, the available data suggests that the supervised ES, in combination with the motivating influence of the TC, were crucial for the reported changes in some patients. In contrast to the positive impact reported by the patients, the GPs did not observe any noticeable changes, and only a few sports therapists observed a positive impact on patients’ exercise behavior. This is not unexpected given the previous statements of the GPs, that some forgot which patients had received the intervention. In addition, the FR, which were supposed to present the changes in medical parameters throughout the intervention, were rarely discussed. An evaluation of the intervention’s impact by GPs seems to have been lacking in most cases. On the other hand, the sports therapists had a significantly closer contact with the patients. Therefore, they may have been in a better position to assess the positive impact. However, these statements are also based on subjective impressions from supervised ES. Especially in the later stages, the contact between the PSG and the sports therapist decreased significantly, so that these observations must be interpreted with caution. Another reason for the lack of observed impact by the GPs may be the frequency of PSG meetings. Group meetings in P-SUP were held once a week, unless the PSG met on additional days. Therefore, the amount of time spent in the weekly ES was probably not sufficient to make a noticeable difference, as the WHO recommends at least 150 min of physical activity per week [81]. Although the patients were encouraged to exercise beyond the weekly ES, the extent to which they did so was not assessed. In summary, data from the quantitative process evaluation of P-SUP need to be further considered to assess the adherence of the different components. Combined with data from the quantitative efficacy evaluation, more precise conclusions about the impact of the different components can be drawn.

### Limitations

In this study, focus group interviews and expert interviews were conducted to gain insight into the implementation of P-SUP. On the patient side, both active participants and dropouts who left the intervention for various reasons were recruited. Efforts were made to select dropouts who had attended at least one PSG meeting before discontinuing participation, ensuring exposure to all program components. This subgroup was examined to elucidate reasons for their withdrawal. Therefore, dropouts were asked about their experiences in the same way as the other patients. Whether patients dropped out due to dissatisfaction or for other reasons was clarified by the patients themselves during the focus groups. However, early withdrawals may have led to a more negative overall program rating based on initial impressions, without a comprehensive insight into the components. Nevertheless, including these patients seems important to gather valuable insights from potentially dissatisfied participants, mitigate attrition bias, and achieve a comprehensive understanding of the intervention. The low participation rates among patients (45%), GPs (13%) and sports therapists (41%) entail a risk of selection bias, as potentially motivated individuals may have been more inclined to participate. This may have biased the results less representative, although this assertion remains speculative, as only patients provided reasons for their refusal during direct telephone contact. The IST withdrew without providing reasons, or in most cases, did not respond to the invitation letters. However, no further recruitment was pursued as the number of focus group interviews and expert interviews has reached saturation [42]. A more balanced ratio of dropouts to active patients may help to avoid this problem in future studies. In this study, however, only dropouts who attended at least one PSG meeting were included. This reduced the potential sample size because some patients dropped out before the first group meeting due to restrictions imposed by the Covid 19 pandemic and never attended a group meeting. Inclusion of dropouts without active participation may have provided further insight into potential barriers that occurred regardless of program implementation.

On the IST side, almost all professions were included in this study. Only the university hospital staff, which was responsible for the intervention organization, was not interviewed. Because the interviewers and the university hospital staff were involved in the design of P-SUP and knew each other personally, there was a risk of subject bias. Moreoever, given the interviewers’ engagement in the development of the P-SUP intervention, there is a potential risk for interviewer bias, stemming from their formulation of questions and their reaction to respondents’ answers, which may influence participants’ responses. However, precautions were implemented to mitigate this bias by ensuring that interviewers had no prior contact with participants before conducting the interviews. Hiring an external organization to conduct the interviews could have avoided this limitations, and potentially important information from the university hospital staff might have been included in this process evaluation.

For organizational reasons, IST were interviewed by expert interviews, and patients were interviewed by focus group interviews. Focus group interviews can help to confirm, extend or enrich understanding and provide additional information [38]. The interaction between participants often leads to lively discussion and can therefore facilitate the collection of more in-depth data. However, focus group interviews are subject to social desirability bias due to the presence of other participants and an interviewer. This could have led to an overestimation of positive and negative experiences and might have reduced the heterogeneity of responses [82].

## Conclusions

Despite some limitations, this process evaluation provides useful information about patients’ and IST experiences with the implementation of P-SUP. Multiprofessional interventions such as P-SUP seem feasible to implement in DMP care and appear to be valuable for patients and IST. In general, peer support might be a valuable approach for patients who have difficulties making lifestyle changes. However, more frequent supervision and regular feedback are crucial for the implementation of PSGs. In this context, TC in particular has been a promising method for patient motivation and regular feedback. The digital components of P-SUP (DEE, WP) received mixed evaluations due to the technical resources of the target group. To save resources for IST and patients, comparable digital interventions should continue to be offered and thematically adapted to the interests of the target group. These findings can be used to guide researchers and IST in future studies to improve the feasibility and effectiveness of interventions in different contexts.

## Electronic supplementary material

Below is the link to the electronic supplementary material.

Supplementary Material 1

Supplementary Material 2

## Data Availability

The dataset used and/or analyzed during the current study are available from the corresponding author on reasonable request.

## Abbreviations

CHD: Coronary Heart Disease
DEE: Digital Expert Education Classes
DMP: Disease Management Program
ES: Exercise Sessions
FR: Feedback Reports
GPs: General Practitioners
IST: Implementing Stakeholders
PSG: Peer Support Group
SPO framework: Structure-Process-Outcome Framework
T2DM: Type 2 Diabetes Mellitus
TC: Telephone Coaching
WP: Web Portal
