# Publisher Correction: Patients’ and stakeholders’ experiences of a personalized self-management SUPport program (P-SUP) for patients with type 2 diabetes mellitus and/or coronary heart disease: a qualitative process evaluation

**DOI:** 10.1186/s12889-024-20247-9

**Published:** 2024-10-23

**Authors:** Maximilian Scholl, Jessica Amerkamp, Chloé Chermette, Friederike Frank, Christian Funke, Lisa Giesen, Viviana Haas, Martina Heßbrügge, Lucas Küppers, Larisa Pilic, Frank Vitinius, Bianca Biallas

**Affiliations:** 1 Institute of Movement Therapy and Movement-oriented Prevention and Rehabilitation, German Sports University Cologne, Cologne, Germany; 2 Institute of Psychology, German Sports University Cologne, Cologne, Germany; 3 Institute for Digitalization and General Medicine, Medical Faculty, RWTH Aachen University, Aachen, Germany; 4 Center for Rare Diseases Aachen (ZSEA), Medical Faculty, RWTH, Aachen University, Aachen, Germany; 5 IInstitute of General Practice, Heinrich Heine University Düsseldorf, Düsseldorf, Germany; 6 Institute of Health Economics and Clinical Epidemiology, University Hospital Cologne, Cologne, Germany; 7 Institute of General Practice, University Duisburg-Essen, Essen, Germany; 8 Institute of Family Medicine and General Practice, University of Bonn, Bonn, Germany; 9 Institute of General Practice, Faculty of Medicine, University of Cologne, Cologne, Germany; 10 Department of Psychosomatics and Psychotherapy, Faculty of Medicine, University Hospital and University of Cologne, Cologne, Germany; 11 Department of Psychosomatic Medicine, Robert-Bosch Hospital, Stuttgart, Germany

**Publisher Correction: BMC Public Health (2024) 24:2566**

10.1186/s12889-024-20034-6

The original publication of this article contained an erroneous subtitle due to an error in the publication process. The original article has been updated to remove the subtitle.

