# Supplementary material for: Patients’ and stakeholders’ experiences of a personalized self-management SUPport program (P-SUP) for patients with type 2 diabetes mellitus and/or coronary heart disease: a qualitative process evaluation
Source: BMC Public Health. 2024 Sep 19;24:2566. doi: 10.1186/s12889-024-20034-6 (PMC11414288; doi:10.1186/s12889-024-20034-6)
Supplement: Supplementary file 1 — Supplementary Material 1 [file 12889_2024_20034_MOESM1_ESM.pdf]

P-SUP interview guide for focus group interviews

Patients

Main aspects
1. What has been your experience with the group meetings?
2. What has been your experience with the exercise sessions?<br>2.1. Experiences with supervised sessions with a sports therapist?<br>2.2. Experiences with self-managed sessions without a sports therapist?
3. What has been your experience with the digital expert education classes?
4. What has been your experience with the web portal?
5. What has been your experience with the telephone coaching? [1]
6. What has been your experience with the feedback reports?
7. What has been your experience with the role of the PSG Leader? [2]
8. What has been your experience with the P-SUP organization?<br>8.1. Experiences with the infrastructure<br>8.2. Experiences with the intervention staff<br>8.3. Experiences with the communication
9. How would you rate the materials you received?
10. To what extent would you say that participating in P-SUP has had an impact on you?
11. What were your expectations for P-SUP and how did they compare with your experience?
12. Where do you think P-SUP needs to improve in general?

[1] Question for focus group interviews with patients, who received telephone coaching.
[2] Question for focus group interviews with patients, who have been PSG Leaders.

P-SUP interview guides for telephone based expert interviews

Sports therapists

**Main aspects**
1. What has been your experience with the group meetings?
2. What has been your experience with the exercise sessions?<br>2.1. Experiences with supervised sessions under your supervision?<br>2.2. Experiences with self-managed sessions without your supervision?
3. How would you rate the materials you received?
4. What feedback have you received from patients about other P-SUP components?
5. What has been your experience with the P-SUP organization?<br>5.1. Experiences with the infrastructure<br>5.2. Experiences with the intervention staff<br>5.3. Experiences with the communication
6. To what extent would you say that participating in P-SUP has had an impact on patients?
7. What were your expectations for P-SUP and how did they compare with your experience?
8. Where do you think P-SUP needs to improve in general?

General practitioners

**Main aspects**
1. What has been your experience in implementing P-SUP in your practice?
2. What has been your experience with the patient enrollment?
3. How would you rate the materials you received?
4. What has been your experience with the feedback reports?
5. What feedback have you received from patients about other P-SUP components?
6. What has been your experience with the P-SUP organization?<br>6.1. Experiences with the infrastructure<br>6.2. Experiences with the intervention staff<br>6.3. Experiences with the communication
7. To what extent would you say that participating in P-SUP has had an impact on patients?
8. What were your expectations for P-SUP and how did they compare with your experience?
9. Where do you think P-SUP needs to improve in general?

Experts

**Main aspects**
1. What has been your experience with the group meetings?
2. What has been your experience with the digital expert education classes?
3. How would you rate the choice of topics for the digital expert education classes?
4. How did patients respond to the topics of the education classes?
4. What feedback have you received from patients about other P-SUP components?
5. What has been your experience with the P-SUP organization?<br>5.1. Experiences with the infrastructure<br>5.2. Experiences with the intervention staff<br>5.3. Experiences with the communication
6. Did you geht any feedback from patients, that participating in P-SUP has had an impact on them?
7. What were your expectations for P-SUP and how did they compare with your experience?
8. Where do you think P-SUP needs to improve in general?

Telephone coaches

**Main aspects**
1. What has been your experience with telephone coaching?
2. How would you rate the tools of the telephone coaching?
3. How did patients respond to the contents of the telephone coaching?
4. What feedback have you received from patients about other P-SUP components?
5. What has been your experience with the P-SUP organization?<br>5.1. Experiences with the infrastructure<br>5.2. Experiences with the intervention staff<br>5.3. Experiences with the communication
6. Did you geht any feedback from patients, that participating in P-SUP has had an impact on them?
7. What were your expectations for P-SUP and how did they compare with your experience?
8. Where do you think P-SUP needs to improve in general?
