# Supplementary material for: Patients’ and stakeholders’ experiences of a personalized self-management SUPport program (P-SUP) for patients with type 2 diabetes mellitus and/or coronary heart disease: a qualitative process evaluation
Source: BMC Public Health. 2024 Sep 19;24:2566. doi: 10.1186/s12889-024-20034-6 (PMC11414288; doi:10.1186/s12889-024-20034-6)
Supplement: Supplementary file 2 — Supplementary Material 2 [file 12889_2024_20034_MOESM2_ESM.pdf]

1) Peer-Support-Groups (PSGs)

Due to time constraints and a lack of resources in primary care, the delivery of self-management education and support has been shifting to a peer support model, i.e. support from people with the same or a similar disease (Fisher et al., 2014). Peer Support is an effective way to improve patients' medical parameters and positively influence their lifestyle. (Gatlin et al., 2017; Patil et al., 2018). Compared to support from professionals, peer support has the advantage of being cost-effective, non-hierarchical, providing long-term emotional support and potentially sharing similar life experiences.

In this study, PSGs are composed of DMP patients with T2DM and/or CHD who are enrolled in the intervention by their general practitioners. The PSGs are organized by a leading group member (i.e. the PSG Leader) who also has at least one of these conditions and who has been trained for this task before the start of the intervention *(for more detailed information on the content of this training, see the study protocol by Konerding et al., 2021)*.

PSGs participate in weekly *exercise sessions*. A total of 14 exercise sessions are supervised by a sports therapist. At the beginning of the intervention, these sessions are frequently supervised. The frequency of supervised sessions decreases over time. The goal of the supervised sessions is to develop an exercise plan with the PSG and to prepare the group for self-managed exercise sessions without the supervision of a sports therapist. In order to standardize these sessions, sports therapists and PSGs were provided with a target-group-specific exercise manual that included warm-up and cool-down rituals, various exercise suggestions, circuit training, and a resistance band. Sports therapists were free to modify these exercises according to the needs and physical abilities of the PSG. The PSG can choose to do the exercises indoors or outdoors. As much as possible, the groups should organize the location themselves.

A total of eight *digital expert education* classes on disease and lifestyle related subjects are offered to the patients. These classes are delivered online via a videoconferencing tool by physicians, nutritionists, psychologists, sports scientists and health scientists on the topics of medicine, nutrition, communication/group dynamics, and motivation. Patients conduct these digital classes from home using their own devices. The PSGs are free to choose additional topics based on their own interests, such as physical activity, living with chronic disease, or psychological aspects of chronic disease. Additionally, the PSGs were given the option to use some of these weekly appointments as round tables to discuss topics of interest (see Figure for schedule).

Figure: Schedule for PSGs

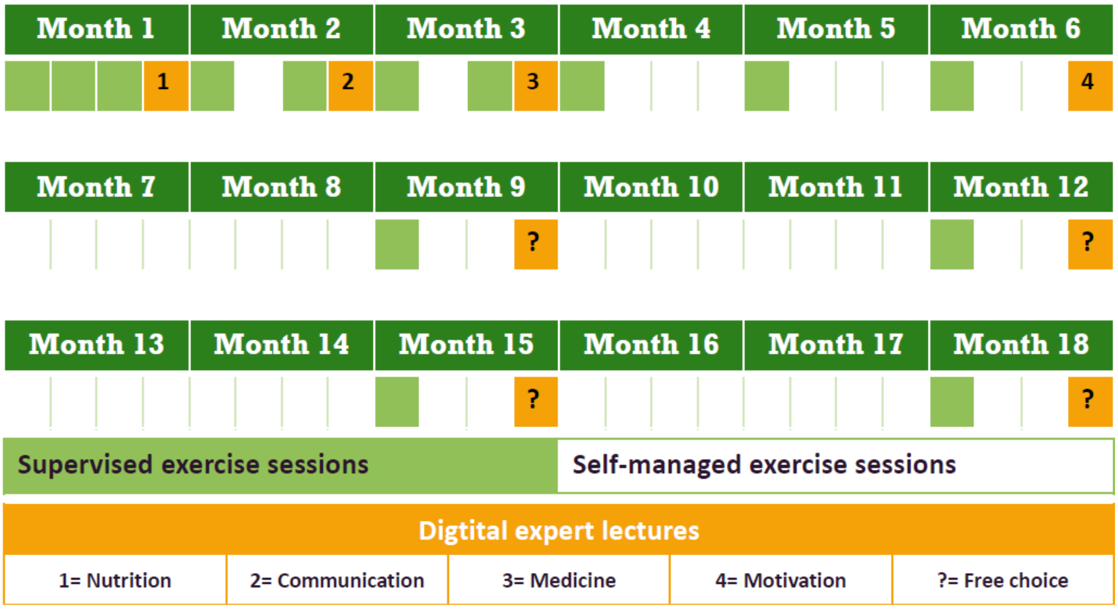

2) Telephone coaching (TC)

TC is a way to deliver health coaching by phone, which allows health care professionals reaching even rural areas and impaired patients. TC has the potential to help patients change their health behavior (Linden et al., 2010), social functioning, self-efficacy, patient activation and perceived health (Linden et al., 2010; Sacco et al., 2009). Furthermore, studies show that TC can reduce the hospitalization rates and healthcare costs (Wennberg et al., 2010). For more information see TC concept paper by Gawlik et al., 2023.

In this study, TC is offered to a subsample of the study intervention with low health literacy, as measured by the Brief Health Literacy Scale (Sand-Jecklin & Coyle, 2014), and/or low activation level, as measured by the Patient Activation Measure (Brenk-Franz et al., 2013; Hibbard et al., 2004). TC aims to promote physical activity and healthy eating and consists of 13 sessions of 20 to 30 minutes each (Eakin et al., 2007; see Table).

Table: Schedule for Telephone coaching

Months	1	2	3	4	5	6	7	8	9	10	11	12	13	14	15	16	17	18
Number of calls	2	2	1	1	1	1		1		1		1		1				1
Duration (in min.)	30	20	20	20	20	20		20		20		20		20				30

3) Patient Feedback Reports

Ongoing feedback is an essential intervention component to promote healthy lifestyles (Konerding & Szel, 2021). In this study, feedback reports provide patients with information on whether their values have worsened, stayed the same, improved, or are within the normal range. These reports contain routine data and graphically show the development of medical parameters such as HbA1c, blood pressure, weight and cholesterol over time. These reports are sent to the GPs, who send them to their patients and can be used as a basis for discussing a patient's state of health at DMP check-ups.

4) Browser-based web portal

Digital behavior change interventions (DBCIs) represent a novel and scalable approach to delivering tailored behavior change interventions (Forberger et al., 2017). DBCIs are interventions that use digital technology to promote and support behavior change that maintains or improves health by preventing and managing health problems and can be deliverd, for example, through computer programs, websites, mobile apps, or wearable devices (Hekler et al., 2016). As such, DBCIs have the potential to improve outcomes, reduce costs and enhance the patient experience (Michie et al., 2017).

In this study, a web portal is accessible to all participants in the intervention group and provides content on exercise, nutrition, and motivation. It includes:

- A manual with suggestions for the exercise sessions with the PSG or alone
- 10 at-home exercise videos (see Figure)
- A variety of healthy recipes (see Figure)
- Downloadable food diaries
- Evidence-based information on medical basics of T2DM and CHD, physical activity, nutrition, and self-motivation
- A 12-week coaching to help patients manage their own health-related behaviors.

Figure: Exercise videos

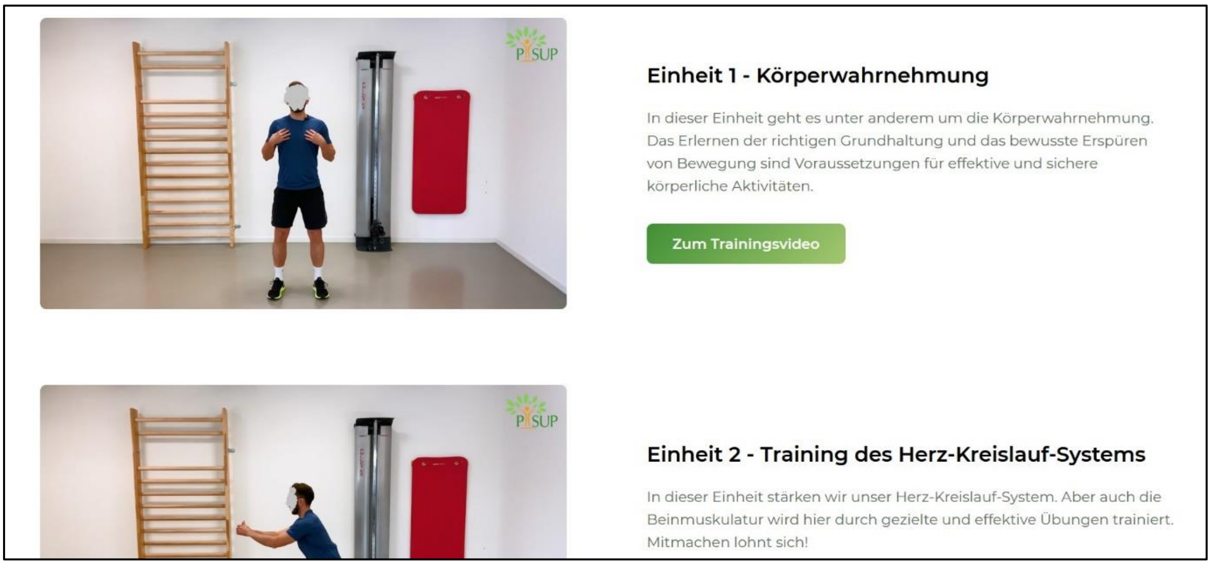

Figure: Recipes

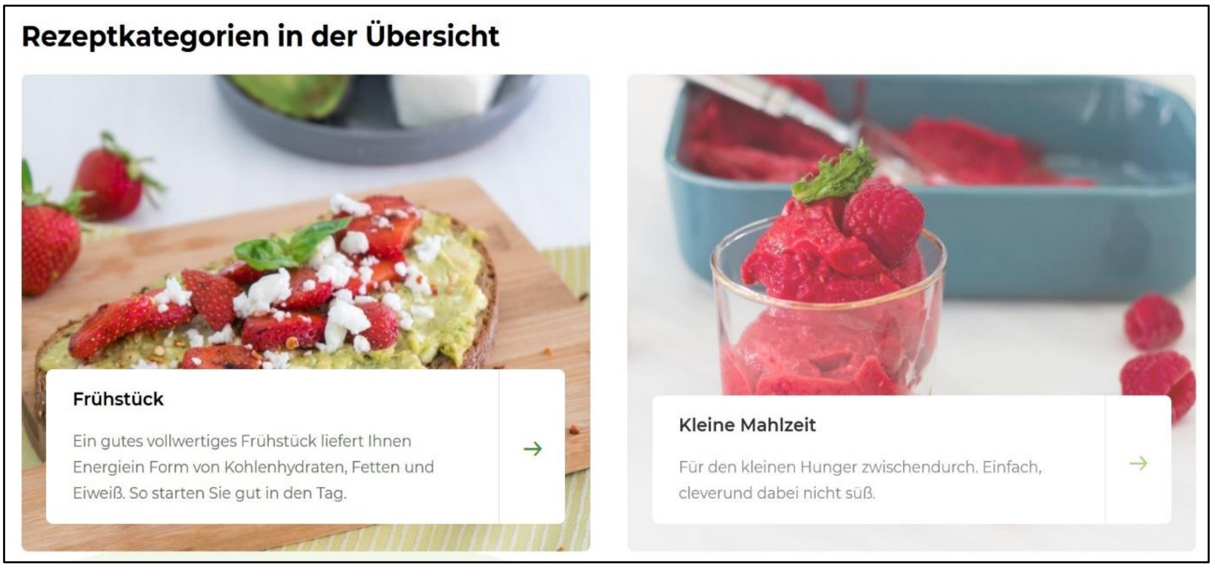

The 12-week coaching includes 12 learning modules on motivation and behavior change, using behavior change techniques (BCTs) based on the Health Action Process Approach (HAPA) model (Schwarzer, 1992). More detailed information about the learning modules is provided in the following Table.

Personalised Self-management **SUP**port Programme **(P-SUP)**

Table: Learning modules of the 12-week coaching

Week	Module title	Module aim	Module content
**1**	Activity check	- Understanding the effects of physical (in)activity and introducing WHO recommendations<br>- Determine one's own physical activity through self-assessment<br>- Comparison of WHO minimum requirements and actual status	- Survey of daily and sports-related activity based on the GPAQ<br>- Individual feedback on current every day and exercise-related activity
**2**	Decision balance	- Recognizing and becoming aware of the perceived advantages and disadvantages of a behavior change<br>- Mitigate/reduce one (or more) disadvantages<br>- Securing one (or more) advantages	- Gathering perceived advantages and disadvantages of a behavior change<br>- Mitigate/reduce one (or more) disadvantages<br>- Securing one (or more) advantages
**3**	My exercise motivation	- Knowledge transfer on different motivational models<br>- Self-diagnosis of regulation mode<br>- Stimulating the internalization process	- Providing information about different motivational modes<br>- Assessment of motivation to move (using the SSK scale)<br>- Visualization of the result<br>- Individual feedback and stimulation of the internalization process
**4**	Exercise type	- Generating self-concordant exercise characteristics	- Defining which characteristics the activity should have (exercise preference)<br>- Create a personal exercise-profile
**5**	My exercise goal	- Support in setting a self-concordant goal	- Explaining the meaning of goals<br>- Supporting the development of a self-concordant goal<br>- Applying the SMART technique to goals
**6**	Imagination of the exercise goal	- Consolidating the previously defined self-concordant goal	- Introduction to visualization techniques<br>- Exercise to visualize one's own movement goal
**7**	Exercise planner	- Strengthening the ability to create one's own exercise / health plan<br>- Long-term integration of exercise sessions	- Introduction to making plans<br>- Collecting concrete exercise ideas (what, where, when, how often, with whom)<br>- Planning the concrete exercise ideas into a weekly plan
**8**	Health diary	- Documentation of current health behavior<br>- Increasing awareness of one's own behavior	- Introduction to self-monitoring<br>- Choice of diary categories (exercise + diet / mood / sleep)<br>- Evaluating the health diary
**9**	Establishing a routine	- Daily integration of healthy habits<br>- Establishing an automatized health behavior	- Clarifying the function of routines<br>- Choosing an appropriate routine<br>- Planning the daily routine
**10**	My bridges	- Becoming aware of one's own barriers<br>- Developing personal solution strategies	- Learn about different types of barriers<br>- Identifying personal barriers<br>- Developing appropriate solution strategies
**11**	Self-reflection and deepening	- Analyzing needs and reinforcing a behavioral change strategy	- Encourage self-reflection on areas of behavior change where problems/needs still exist.<br>- Targeted repetition of a previous module
**12**	My future self-management	- Long-term planning of an active lifestyle	- Encourage reflection on which behavioral changes are appropriate and feasible.<br>- Developing a future, long-term physical activity plan<br>- Complete the motivational modules

References

Brenk-Franz, K., Hibbard, J. H., Herrmann, W. J., Freund, T., Szecsenyi, J., Djalali, S., ... & Gensichen, J. (2013). Validation of the German version of the patient activation measure 13 (PAM13-D) in an international multicentre study of primary care patients. *PloS one*, *8*(9), e74786. https://doi.org/10.1371/journal.pone.0074786

Eakin, E. G., Lawler, S. P., Vandelanotte, C., & Owen, N. (2007). Telephone interventions for physical activity and dietary behavior change: a systematic review. *American journal of preventive medicine*, *32*(5), 419-434. https://doi.org/10.1016/j.amepre.2007.01.004

Fisher, E. B., Coufal, M. M., Parada, H., Robinette, J. B., Tang, P. Y., Urlaub, D. M., ... & Xu, C. (2014). Peer support in health care and prevention: cultural, organizational, and dissemination issues. *Annual review of public health*, *35*, 363-383.

Forberger, S., Bammann, K., Bauer, J., Boll, S., Bolte, G., Brand, T., ... & Zeeb, H. (2017). How to tackle key challenges in the promotion of physical activity among older adults (65+): the AEQUIPA network approach. *International journal of environmental research and public health*, *14*(4), 379.

Gatlin, T. K., Serafica, R., & Johnson, M. (2017). Systematic review of peer education intervention programmes among individuals with type 2 diabetes. *Journal of Clinical Nursing*, *26*(23–24), 4212–4222. https://doi.org/10.1111/jocn.13991

Gawlik, A., Nacak, Y., Kleinert, J., Konerding, U., & Vitinius, F. (2023). Theoretical Derivation of a Telephone-Based Health Coaching Intervention for Promoting Physical Activity and Healthy Nutrition. *International Journal of Environmental Research and Public Health*, *20*(13), 6271. https://doi.org/10.3390/ijerph20136271

Hekler, E. B., Michie, S., Pavel, M., Rivera, D. E., Collins, L. M., Jimison, H. B., ... & Spruijt-Metz, D. (2016). Advancing models and theories for digital behavior change interventions. *American journal of preventive medicine*, *51*(5), 825-832.

Hibbard, J. H., Stockard, J., Mahoney, E. R., & Tusler, M. (2004). Development of the Patient Activation Measure (PAM): conceptualizing and measuring activation in patients and consumers. *Health services research*, *39*(4p1), 1005-1026. https://doi.org/10.1111/j.1475-6773.2004.00269.x

Konerding, U., Redaèlli, M., Ackermann, K., Altin, S., Appelbaum, S., Biallas, B., Bödecker, A.-W., Botzenhardt, S., Chermette, C., Cichocki, M., Dapper, I., Dehnen, K., Funke, C., Gawlik, A., Giesen, L., Goetz, J., Graf, C., Hagen, B., Heßbrügge, M., … Stock, S. (2021). A pragmatic randomised controlled trial referring to a Personalised Self-management SUPport Programme (P-SUP) for persons enrolled in a disease management programme for type 2 diabetes mellitus and/or for coronary heart disease. *Trials*, *22*(1), 659. https://doi.org/10.1186/s13063-021-05636-4

Konerding, U., & Szel, C. (2021). Promoting physical activity in persons with type 2 diabetes mellitus: A systematic review of systematic reviews. *Patient Education and Counseling*, *104*(7), 1600–1607. https://doi.org/10.1016/j.pec.2020.12.011

Michie, S., Yardley, L., West, R., Patrick, K., & Greaves, F. (2017). Developing and evaluating digital interventions to promote behavior change in health and health care: recommendations resulting from an international workshop. *Journal of medical Internet research*, *19*(6), e232.

Linden, A., Butterworth, S. W., & Prochaska, J. O. (2010). Motivational interviewing-based health coaching as a chronic care intervention. *Journal of Evaluation in Clinical Practice*, *16*(1), 166–174. https://doi.org/10.1111/j.1365-2753.2009.01300.x

Patil, S. J., Ruppar, T., Koopman, R. J., Lindbloom, E. J., Elliott, S. G., Mehr, D. R., & Conn, V. S. (2018). Effect of peer support interventions on cardiovascular disease risk factors in adults with diabetes: A systematic review and meta-analysis. *BMC Public Health*, *18*(1), 398. https://doi.org/10.1186/s12889-018-5326-8

Sacco, W. P., Malone, J. I., Morrison, A. D., Friedman, A., & Wells, K. (2009). Effect of a brief, regular telephone intervention by paraprofessionals for type 2 diabetes. *Journal of Behavioral Medicine*, *32*(4), 349–359. https://doi.org/10.1007/s10865-009-9209-4

Sand-Jecklin, K., & Coyle, S. (2014). Efficiently assessing patient health literacy: the BHLS instrument. *Clinical Nursing Research*, *23*(6), 581-600. https://doi.org/10.1177/1054773813488417

Schwarzer, R. (1992). Self-efficacy in the adoption and maintenance of health behaviours: Theoretical approaches and a new model. *Self-efficacy: Thought control of action*, *217*, 242.

Wennberg, D. E., Marr, A., Lang, L., O'Malley, S., & Bennett, G. (2010). A randomized trial of a telephone care-management strategy. *The New England Journal of Medicine*, *363*(13), 1245–1255. https://doi.org/10.1056/NEJMsa0902321
